# Management of Forgotten Ureteral Stents: Relationship Between Indwelling Time and Required Treatment Approaches

**DOI:** 10.4274/balkanmedj.2015.1562

**Published:** 2017-08-04

**Authors:** Hacı Polat, Mehmet Özgür Yücel, Mehmet M. Utangaç, Can Benlioğlu, Alper Gök, Ali Çift, Bedreddin Kalyenci, Uğur Lök, Umut Gülaçtı

**Affiliations:** 1 Department of Urology, Adıyaman University School of Medicine, Adıyaman, Turkey; 2 Department of Urology, Dicle University School of Medicine, Diyarbakır, Turkey; 3 Department of Emergency, Adıyaman University School of Medicine, Adıyaman, Turkey

**Keywords:** disease management, foreign bodies, urinary catheters, urolithiasis

## Abstract

**Background:**

Double-J stents are widely used in urology practice, and removal of these stents can sometimes be forgotten.

**Aims:**

To investigate whether indwelling time of double-J stent can predict which treatment modality is required for removal of the stent from the body.

**Study Design:**

A multicentre, retrospective observational study.

**Methods:**

The data of 57 patients who were treated for forgotten ureteral stents between January 2007 and December 2014 were evaluated retrospectively. Patients were classified into four groups according to indwelling time of the stents: 6-12 months, 13-24 months, 25-36 months, and >36 months. Encrustation and associated stone burden of the stents were evaluated with non-contrast stone protocol computerised tomography.

**Results:**

Patients were classified according to their duration of the stent indwelling time. Simple cystoscopic stent retrieval was performed in 71.4% of patients in the 6-12 months group, 44% of patients in the 13-24 months group, 6.2% of patients in the 25-36 months group, and 11.1% of patients in the >36 months group. A percutaneous or open surgery was required in no patients with an indwelling time of double-J stent shorter than 30 months.

**Conclusion:**

Transurethral and/or percutaneous combined endo-urological approaches are usually sufficient for the removal of forgotten double-J stents. Transurethral procedures are sufficient for the treatment of patients with double-J stent indwelling times less than 30 months.

Double-J stents (DJSs) are widely used in urological practice for various reasons. The removal of these stents can sometimes be forgotten. Unfortunately, management of these forgotten stents is difficult, complicated, time consuming, and costly, both for patients and for physicians. Retention of ureteral stents, which usually occurs due to poor compliance of the patient, is not rare, especially in regions of low socio-cultural development.

Encrustation on stents is a well-established complication of retained DJS in the urinary tract (1). The amount of encrustation on a stent is associated with its indwelling time in the body (2).

As DJS indwelling time increases, more complicated operations are required for removal from the body. However, probably due to an insufficient number of patients, to our knowledge no study is yet available in the literature that directly addresses this issue. In this study, we tested the hypothesis that longer indwelling times require more complicated surgery in the largest patient series assessed so far. By looking at the DJS indwelling time, we investigated whether the treatment modality required for its removal from the body is predictable or not.

## MATERIALS AND METHODS

After local ethics committee approval, we retrospectively evaluated treatment modalities applied to forgotten ureteral stents (FUS) in 57 patients at two neighbouring Turkish referral urology departments where urinary tract stone surgery was commonly performed. Informed consent was obtained from all participants included in the study. Patients were treated between January 2007 and December 2014. The indwelling time of DJSs was calculated from the time of insertion to the time of removal. Patient records from earlier years were excluded from the study because some of the current therapeutic modalities, such as retrograde intrarenal surgery (RIRS), were not being used at that time. Patients whose DJS indwelling time was less than six months were excluded from the assessment. Two patients with bilateral DJS were excluded from the study due to the difficulty of their evaluation.

Urine culture, serum creatinine, and white blood cell counts were evaluated in all patients. Patients with a positive urine culture were treated preoperatively, according to the culture antibiogram. Before any urological intervention, urine culture negativity was obtained from all patients. In all patients, ultrasonography (USG) and serum creatinine were examined routinely at one to three months postoperatively.

Indwelling DJSs in the body were determined using a plain x-ray of the kidneys, ureters, and bladder [plain X-ray of the kidneys, ureters, and bladder (KUB graphy)]. Stent encrustation and associated stone burden were evaluated using non-contrast enhanced stone protocol computerised tomography (CT). A Tc99m dimercaptosuccinic acid scan was performed for patients with presumptive renal parenchymal damage based on CT.

The stone burden adjacent to the encrusted stent was calculated using the following formula: stone burden= length × width of calcification surrounding stent on CT (3,4). Based on the calculated area on CT, the encrustation was graded as light (less than 100 mm2), moderate (100 to 400 mm^2^), or severe (greater than 400 mm^2^).

### Statistical analysis

Analysis of variables was performed using SPSS for Windows, version 11.5 (SPSS Inc., Chicago, IL, USA). The normality of distribution of continuous variables was determined using the Kolmogorov-Smirnov test. The Levene test was used to evaluate the homogeneity of variances. Variables are shown as mean ± standard deviation or median (minimum-maximum), as appropriate. Mean differences among duration of FUS groups were analysed by one-way ANOVA; otherwise, Kruskal-Wallis tests were performed for comparisons of median values. When the p values obtained by Kruskal-Wallis test statistics were statistically significant, Conover’s multiple comparison test was used as pairwise post-hoc test to determine which groups differed from each other. To compare categorical variables, in the RxC contingency tables a likelihood ratio test was used because of the fact that one or more of the cells has an expected frequency of five or less. When the p values from likelihood ratio tests were statistically significant in determining which groups differed from each other, Fisher's exact test was used when one or more of the cells had an expected frequency of five or less. Otherwise, Pearson's chi-square test was performed. The degree of association between the stone burden and time was evaluated using Spearman's rank correlation test. A p value <0.05 was considered statistically significant.

## RESULTS

The reasons for the insertion of stents were urolithiasis (n=47), reconstructive urological interventions (n=7), or instillation for oncological disorders (n=3).

**Figure 1:**
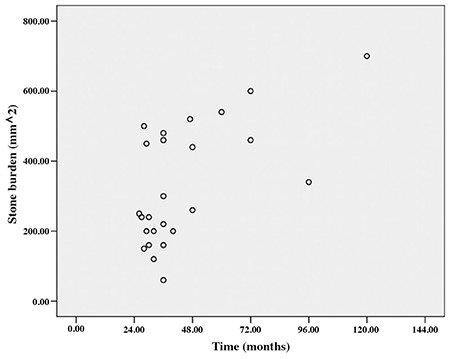
Stone burden was increased in parallel with indwelling time of stent.

**Figure 2. a-d:**
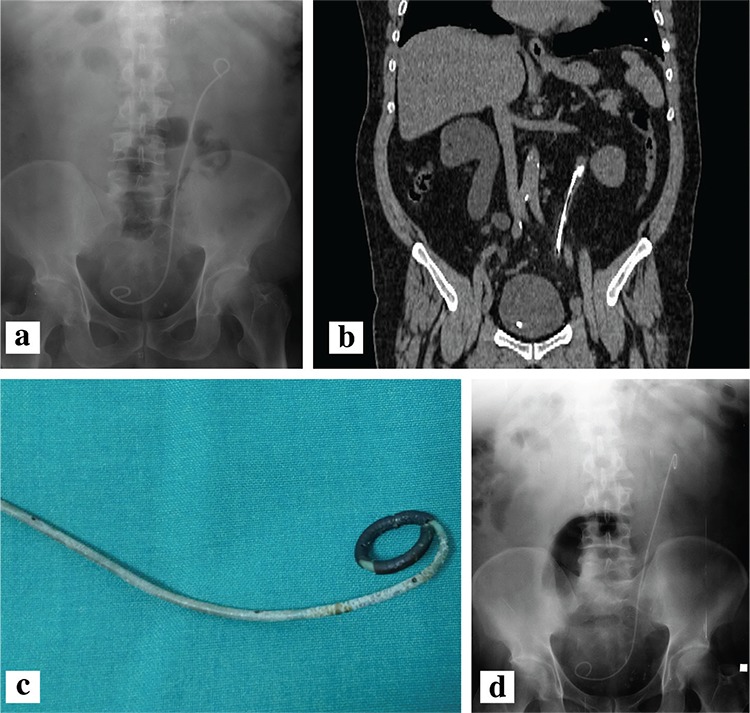
Some images of a patient whose double-J stent had remained in the body for 30 months. Thickened stent due to encrustation (a). Encrustations and stones on computerised tomography, which were not seen in the KUB radiograph (b). Encrustation on the stent; stent was removed by ureteroscopy (c). A new stent inserted in the same patient at the same calibration (6 Fr.) (d). Notice the difference between this new stent and the thickened encrusted stent.

The demographic characteristics of the patients, the amount of encrustation, the length of the hospital stay, and the number of treatment modalities and sessions applied are outlined in Table 1, 2. Severe encrustation was observed on intrarenal (n=7), intravesical (n=4), or intraureteral (n=1) segments of the stents (Table 3). The amount of encrustation and the length of the stay in hospital increased in parallel with the indwelling time of the DJS (Figure 1). The mean hospital stay was two days in the 6-12 months group, three days in the 13-24 months group, four days in the 25-36 months group, and eight days in the >36 months group. The mean (range) number of different treatment modalities applied were 1 (1-2) in the 6-12 months group, 1.5 (1-3) in the 13-24 months group, 2 (1-3) in the 25-36 months group and 2 (1-4) in the >36 months group. The treatment techniques that were applied according to the duration of the FUS are given in Table 1 and Table 4. Only transurethral procedures in addition to extracorporeal shock wave lithotripsy (ESWL), if applied, were used for the removal of DJS in all patients in the 6-12 months and 13-24 months groups. In the 25-36 months group, percutaneous cystolithotripsy had to be applied for four of 16 patients and percutaneous nephrolithotomy (PcNL) for two of 16 patients (Figure 2). In the >36 months group, PcNL had to be applied for four of the nine patients and open surgery (nephrolithotomy and removal of part of the stent) for one patient. Only one patient’s stent was removed using simple cystoscopic stent retrieval (SCSR) after ESWL in each of the 25-36 months and >36 months groups.

## DISCUSSION

Forgotten DJSs are observed in urological practice due to inadequate compliance by the patients or when the patient is insufficiently informed by the physician. These forgotten stents can produce considerable morbidities such as haematuria, urinary tract obstruction, renal failure, and recurrent urinary tract infection (5,6). There is not a specific definition for a forgotten stent. According to our definition, a stent that cannot be removed at the scheduled time is a "forgotten or neglected stent". However, for standardisation purposes, stents that had indwelling times longer than six months were included in this study.

Encrustation of these forgotten DJSs is a serious problem for removal of the stent. Stent encrustation can occur in both infected and non-infected urine. The exact mechanism of encrustation is unclear, but the degree of encrustation is dependent on the indwelling time of the stent (7,8,9). In the present study we found a significant correlation between DJS indwelling time and stent stone burden, as anticipated (Figure 1). In our patients, the most severe encrustation was observed in a patient whose stent had been forgotten for 10 years.

Pre-treatment radiological assessment of patients with FUS is an important issue. According to our experience, KUB graphy and USG are insufficient in this regard, and non-contrast CT should be viewed in these patients. In five patients in our study, KUB graphy viewed for initial diagnosis demonstrated almost no encrustation, but CT revealed significant stone burden in these patients. In addition, in many patients, a greater stone burden could be seen with CT than with KUB graphy. Therefore, we used non-contrast CT to evaluate encrustation and stone burden on the stents. In the evaluation of FUS, the superiority of CT has also been reported in other studies (10,11,12).

Forgotten DJSs with encrustation are a challenging problem for urologists. Multiple urological approaches may be needed because of encrustation and stone burden on the stent. If there is no encrustation on the stent, it can be removed by a simple cystoscopic approach. The longer the indwelling time of the DJS in the body, the greater the amount of encrustation (Table 2, 3). In patients with more delayed DJS, percutaneous and open surgeries were needed as well as these operations. A percutaneous or open surgery was not required in any patient with a DJS indwelling time shorter than 30 months. Open surgery was required only in one patient with a 10-year duration of the stent indwelling.

Previous studies concerning forgotten stents have shown that at 8-12 months after insertion of DJS, encrustations began to form on the external surfaces and inside the lumens of the stents (2,13). Even if there is not apparent encrustation, by reducing the potential stent-mucosal adhesion, ESWL can prevent mucosal injury during stent extraction (14). Most of the patients in this study underwent ESWL as a first treatment, but we now think that, except for cases with an indwelling time of less than one year, ESWL should be applied as the first treatment approach for all forgotten stents.

This study has a few limitations. An important shortcoming of this study is the lack of stone analysis; it may affect the prevention therapy after removal of the DJS. Medical treatments received by patients have not been investigated. The patients' urolithiasis history is not specified. In addition, the types of forgotten stents were not evaluated in this study.

In conclusion, the duration of the indwelling time of forgotten DJSs may predict the required treatment modalities for removal of the stent from the body. If the indwelling time of DJS in the body is less than 12 months, SCSI is sufficient for most patients. If the indwelling time of DJS in body is 13-24 months, transurethral endo-urologic approaches after ESWL are sufficient. Percutaneous or open surgeries may be required only for patients with indwelling time of DJS longer than 30 months. We suggest that 30 months is the threshold time for the removal of forgotten DJS using transurethral approaches.

## Figures and Tables

**Table 1:**
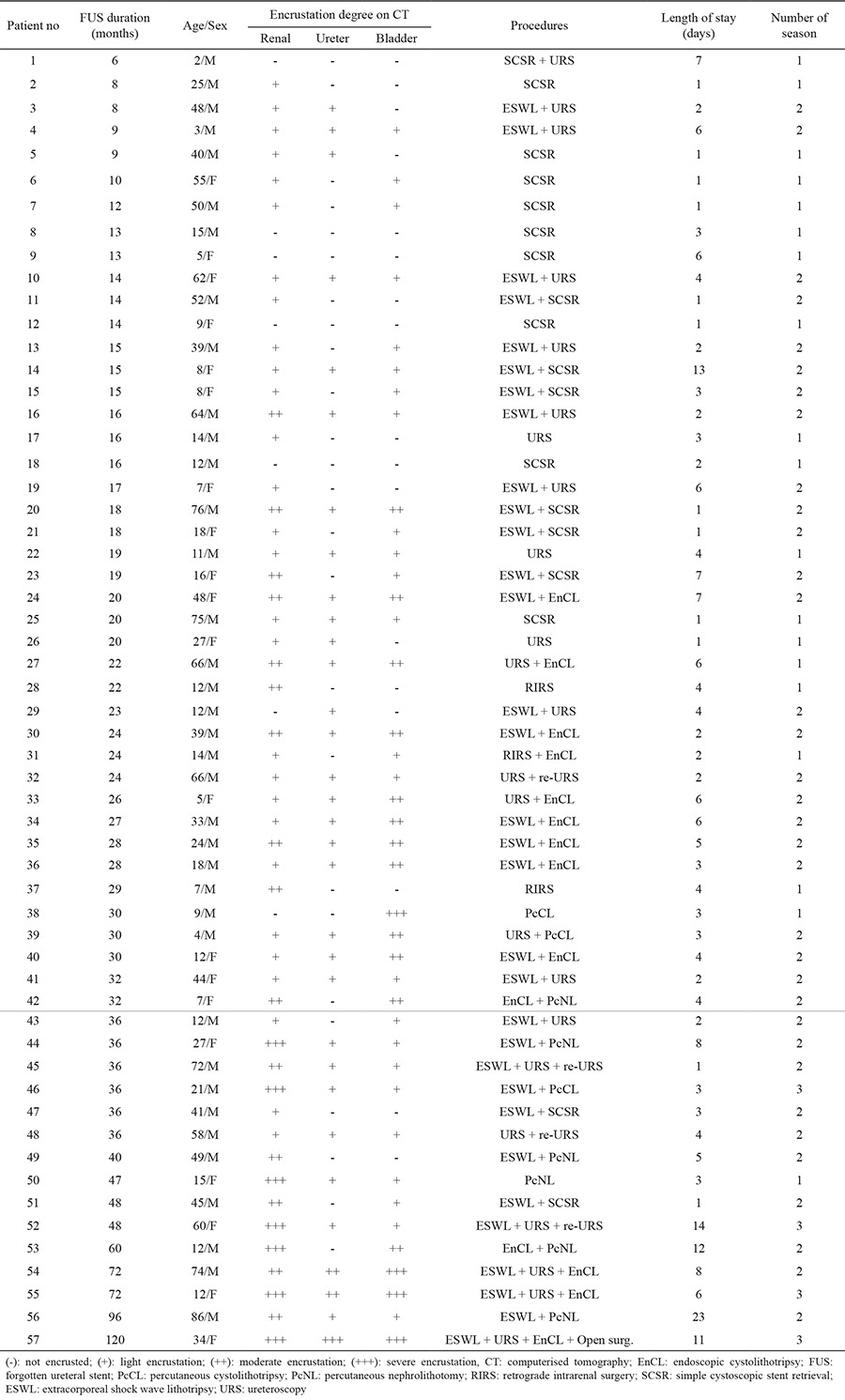
Patients and characteristics

**Table 2:**
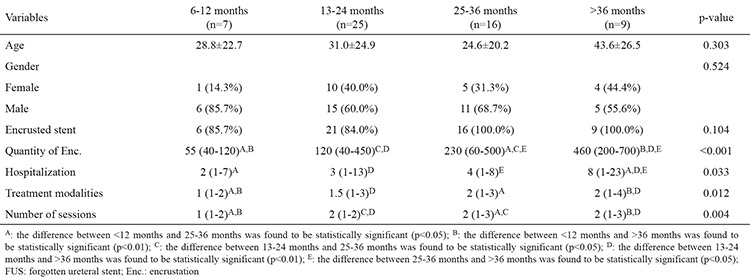
Demographic and clinical characteristics regarding duration of FUS

**Table 3:**
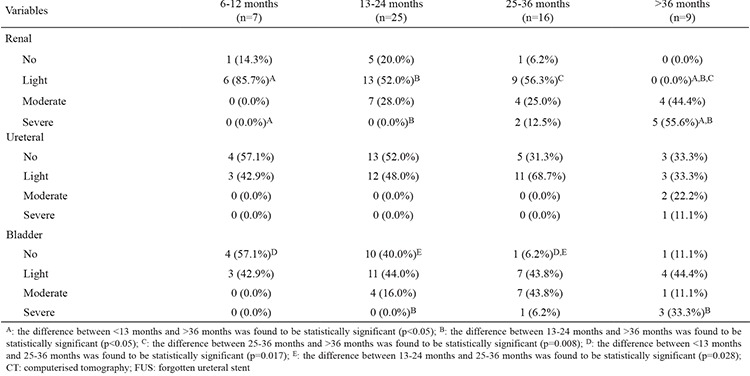
Segment encrustation degree by location on CT regarding indwelling duration of FUS

**Table 4:**
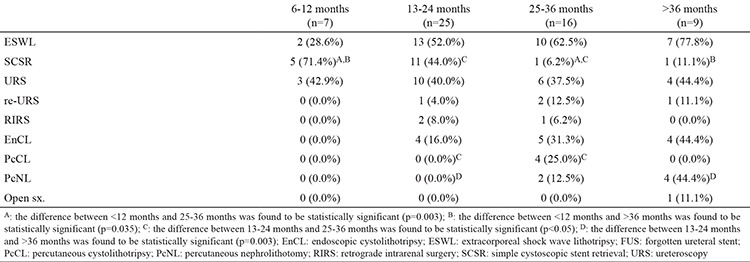
Distributions of surgical types regarding duration of FUS
